# Feasibility, challenges, and solutions for implementing targeted universal tuberculosis testing: perceptions of healthcare professionals in South Africa

**DOI:** 10.1186/s12913-026-14156-3

**Published:** 2026-02-14

**Authors:** Katlego Motlhaoleng, Gary Maartens, Kgomotso Vilakazi-Nhlapo, Jonathan E. Golub, Kate Shearer

**Affiliations:** 1 Department of Medicine, Faculty of Health Sciences, University of Cape Town, Cape Town, South Africa; 2 National Department of Health, Pretoria, South Africa; 3 Center for Tuberculosis Research, Johns Hopkins University, Baltimore, MD USA; 4 Division of Infectious Diseases, Department of Medicine, Johns Hopkins School of Medicine, Baltimore, MD USA; 5 Division of Clinical Pharmacology, K45, Old Main Building, Groote Schuur Hospital, Observatory, Cape Town, 7925 South Africa

**Keywords:** Targeted universal TB testing (TUTT), Drop-Offs, Cascade, PWH

## Abstract

**Background:**

Tuberculosis (TB) remains a leading cause of mortality, particularly among people with HIV (PWH). In South Africa, the targeted universal TB testing (TUTT) strategy was introduced to shift from symptom-based to symptom-agnostic screening to improve early case detection in PWH. However, limited research has explored provider perceptions of the TUTT strategy. We explored healthcare professionals’ perceptions of the introduction, feasibility, challenges, and potential solutions for implementing TUTT.

**Methods:**

We conducted a qualitative study using in-depth interviews with 11 purposively selected healthcare professionals (nurses, program managers, and doctors) involved in integrated TB/HIV care in KwaZulu-Natal Province, South Africa. Interviews were audio-recorded, transcribed, and analysed through inductive thematic analysis.

**Results:**

Four thematic categories with eight overarching themes were identified. TUTT introduction was characterised by largely informal communication, which contributed to variable understanding, while structured dissemination and mentorship supported clearer uptake. TUTT feasibility was shaped by facility capacity, with adequate staffing, diagnostic resources, and workflow organisation enabling smoother integration, whereas under-resourced facilities struggled. Implementation challenges included sputum collection difficulties, especially among asymptomatic PWH, staff shortages, heavy workloads, and fragmented TB/HIV data systems. Proposed solutions centred on expanding access through alternative triage tools such as mobile digital chest X-rays, point-of-care tests, community-based testing, and strengthening provider training, role clarity, and patient education.

**Conclusion:**

TUTT is perceived as a valuable strategy to improve TB detection in PWH, but its success hinges on addressing operational, infrastructural, and patient engagement barriers. Strengthening resources, integrating data systems, and adopting locally tailored, patient-centred approaches are essential to bridge the gap between policy and practice, thereby optimizing TUTT.

**Supplementary Information:**

The online version contains supplementary material available at 10.1186/s12913-026-14156-3.

**Table:** 

Text box 1. Contributions to the literature
• This study addresses a critical evidence gap on how the targeted universal tuberculosis testing (TUTT) strategy has been implemented in real-world settings, drawing on healthcare professionals’ experiences to explore its feasibility and challenges in routine TB/HIV services.
• The findings illuminate how policy communication, provider capacity, diagnostic infrastructure, and fragmented data systems shape TUTT implementation, offering actionable insights for program strengthening.
• By capturing perspectives across multiple levels of the health system, this study contributes to understanding policy adoption dynamics in resource-limited settings and identifies opportunities to enhance the sustainability and scalability of integrated TB/HIV programs.

## Introduction

Tuberculosis (TB), a preventable and curable disease, remains the world’s leading infectious disease killer [1]. In 2024, approximately 10.7 million people fell ill with TB and 1.23 million died, including 150,000 deaths among people with HIV (PWH), many of whom were likely undiagnosed and/or untreated [2]. South Africa carries one of the highest TB burdens worldwide, with an estimated 54,000 TB deaths in 2024, of which approximately 54% (29,000) occurred among PWH [2]. A substantial proportion of these cases present with asymptomatic TB, where individuals have active disease but do not present symptoms, posing a major challenge for symptom-based screening and contributing to delays in diagnosis and treatment initiation [3].

The World Health Organization (WHO) recommended the adoption of symptom-agnostic TB testing strategies to strengthen case detection among populations at highest risk [4, 5]. In line with this guidance, South Africa introduced the targeted universal TB testing (TUTT) strategy in 2021 as part of the second pillar of the National TB Recovery Plan, “Find and Treat” [6]. TUTT marked a shift from the previous approach that tested only individuals with at least one TB symptom (cough, fever, night sweats, and unintentional weight loss) [7]. Under the new strategy, high-risk individuals, including PWH, close contacts of people diagnosed with TB, and individuals with a history of TB within the preceding two years, are eligible for TB testing regardless of symptoms [6, 7].

Despite robust national policies supported by a network of TB experts, implementation challenges persist [8, 9]. Policy roll-out follows a multi-tiered path, from national to provincial structures, to district health services, and finally to frontline settings such as hospitals, clinics, and community health centres [9, 10]. This multi-layered process can dilute fidelity and consistency, creating a gap between policy intent and service delivery [9].

As a recently introduced national policy, TUTT has been the subject of limited research on its implementation in practice [11]. Healthcare professionals play a critical role in identifying people with TB and ensuring timely linkage to care [12]. This study explored their perceptions on the introduction, feasibility, challenges, and potential solutions for improving TUTT implementation.

## Methods

### Study design and setting

We conducted a qualitative study, consisting of in-depth interviews with key informants from different levels of the health department and partner organizations supporting TB/HIV services, including the U.S. President’s Emergency Plan for AIDS Relief (PEPFAR) implementing partners, Global Fund supported partners, and Gates Foundation funded initiatives, in KwaZulu-Natal Province in South Africa. The province carries a high dual burden of TB and HIV [13, 14]. HIV prevalence was estimated at approximately 22% in 2023, with roughly 1.6 million PWH receiving antiretroviral therapy [13]. TB incidence was estimated at 400 cases per 100,000 population in 2022, and more than half of all TB notifications were living with HIV [14].

### Study population

Study participants included a diverse group of healthcare professionals aged ≥ 18 years with at least one year of experience in integrated TB/HIV service provision. Recruitment included individuals from multiple levels of the health department and from implementing partner organizations. These comprised of national health department managers supporting KwaZulu-Natal Province, provincial managers, district and sub-district managers, and facility-based healthcare professionals (nurses and doctors) providing integrated TB/HIV services. Among the nurses, some were facility-based, while others served as implementing partner–supported nurse mentors who worked across clusters of facilities and combined mentorship with direct service delivery. As a result, participants’ roles cut across facilities in diverse geographic contexts within the province, including both urban and rural settings, although rural–urban location was not used as a formal sampling stratum. Healthcare professionals without relevant TB/HIV experience or with less than one year of service in this area were excluded.

### Sampling method and sample size

Purposive sampling was used to identify 15 healthcare professionals with specific expertise in integrated TB/HIV care and management. We anticipated that a sample size of approximately 15–20 participants would be sufficient to reach thematic saturation and obtain a rich understanding of healthcare professionals’ perceptions on the topic. This estimation was informed by prior qualitative studies and the diversity of professional categories included (nurses, doctors, and program managers) [15, 16]. While an initial sample size of 15–20 participants was anticipated and 15 individuals were approached, 4 declined study participation. However, thematic saturation was reached after 10 interviews and an additional interview confirmed saturation. As saturation was achieved, the 4 who declined to participate were not replaced. The final analytic sample, therefore, comprised 11 participants. Participants were identified based on the predefined eligibility criteria through professional networks, recommendations from facility or program managers, and direct engagement during site visits. This approach allowed flexibility to recruit participants across different levels of the health system.

### Interview guide development

An in-depth interview guide was developed specifically for this study to explore healthcare professionals’ experiences with the adoption and implementation of TUTT among PWH. The English version of the guide is provided as a supplementary file (Additional File 1). Broad thematic areas included: (1) introduction of the TUTT policy, (2) extent of adoption and implementation, (3) implementation challenges, and (4) recommendations for improvement. Probing questions were used to elicit detailed responses. Before commencing formal data collection, the guide was piloted with three healthcare professionals working outside KwaZulu-Natal Province (one facility-based nurse, one facility-based doctor, and one district-level program manager), to ensure they were not part of the main study sample. These pilot participants were selected specifically for the pre-testing process to assess clarity, appropriateness, and flow of questions. Data from these pilot interviews were used to improve question phrasing and sequencing and were excluded from analysis.

### Data collection

Interviews were conducted in English between April and July 2024, either in person or virtually via Microsoft Teams, based on participant preference. All interviews were audio-recorded and supplemented by written field notes. Recordings were transcribed verbatim for analysis.

### Data analysis

Descriptive analysis was conducted to summarize participants’ demographic information, while qualitative data were analysed using inductive thematic analysis. Interview transcripts were coded and analysed through a process of constant comparison. Codes and themes were generated inductively, without reliance on predefined categories or frameworks. Initial themes and sub-themes were identified and coded as they emerged from the data. Transcripts were revisited to ensure that any additional themes were identified and incorporated. To enhance analytic credibility, emerging codes and themes from the initial four interview transcripts were discussed with an independent qualitative researcher through iterative peer debriefing, allowing refinement of the coding framework and interpretation. Themes were systematically compared against the study objectives to confirm their alignment with the research question. This iterative process helped to organize and refine the data for analysis. Each theme and sub-theme was analysed in detail, and clear connections between them were identified and described. The analysis involved repeated reading, reflection, and interpretation of the raw data to ensure accuracy and depth. NVivo version 13.0 software was used to support the coding and organization of data during the analysis process.

## Ethical considerations

Ethical approval was granted by the University of Cape Town Human Research Ethics Committee (Ref: 037/2023) and the U.S. Centers for Disease Control and Prevention (Ref: 0900f3eb82189915). Approvals were also obtained from the South African National Department of Health and the KwaZulu-Natal Provincial and District Health Departments. Potential participants were contacted either in person or via email to inform them about the study and request participation in an interview. For in-person interviews, written informed consent was obtained on paper prior to data collection. For virtual interviews conducted via Microsoft Teams, the consent form was shared and completed electronically using Microsoft Forms before the interview. Only participants who returned signed or electronically completed consent forms were included in the study. Anonymity was maintained by assigning unique study IDs and storing identifying information separately from the data. The study was conducted in accordance with the ethical principles outlined in the Declaration of Helsinki and relevant South African national and institutional guidelines for research involving human participants.

## Results

### Participants characteristics

Of the 15 healthcare professionals approached, 11 consented to participate (Table 1). Recruitment challenges were primarily due to participants’ demanding clinical and managerial responsibilities; four individuals declined without providing reasons. The final sample comprised five nurses, four programme managers, and two doctors, representing diverse roles in integrated TB/HIV service delivery. Data saturation was reached by the 10th interview, with one additional interview confirming thematic saturation. Although four individuals declined participation, further recruitment was deemed unnecessary given that saturation had been achieved.

Participants ranged in age from 29 to 55 years (mean: 41 years) and included eight females and three males, reflecting the predominantly female composition of the healthcare workforce. Professional experience in integrated TB/HIV care ranged from 7 to 24 years (mean: 13.4 years), highlighting participants’ substantial expertise in integrated TB/HIV service delivery. Interview durations ranged from 37 to 120 min (mean: 74 min), enabling in-depth exploration of experiences and recommendations regarding TUTT implementation.

**Table 1:** In-Depth interview participants’ characteristics

Participant ID	Health Professional Category	Age	Sex	Years of Service	Interview Duration in Minutes
HP_01	Nurse	40	M	12	84
HP_02	Nurse	53	F	17	60
HP_03	Program Manager	33	F	10	86
HP_04	Program Manager	55	F	15	71
HP_05	Nurse	29	F	7	61
HP_06	Nurse	42	F	15	73
HP_07	Doctor	48	F	24	60
HP_08	Program Manager	40	M	15	120
HP_09	Nurse	31	F	9	72
HP_10	Doctor	42	M	19	80
HP_11	Program Manager	36	F	7	37

HP: Healthcare Professional; Sex: Male (M); Female (F); Age in Years

### Thematic analysis

The analysis explored healthcare professionals’ views across four main categories: the introduction of the TUTT strategy, its feasibility, implementation challenges, and proposed solutions to improve TUTT implementation. Each category contained two overarching themes, supported by sub-themes (Table 2).

**Table 2:** Healthcare professionals’ perceptions of the introduction, feasibility, challenges, and solutions for implementing TUTT

Categories	Themes	Sub-themes
1. The introduction of TUTT	1.1 Communication channels	Informal messaging
		Structured dissemination
	1.2 Capacity building	Training approaches
		Mentorship
4. Feasibility of TUTT	2.1 Facility capacity	Staffing and resource adequacy
		Workflow and operational differences
	2.2 Data infrastructure	Real-time data systems
		Fragmented data collection
7. TUTT implementation challenges	3.1 Operational constraints	Difficulties with sputum collection
		Staff shortages and heavy workloads
	3.2 Patient-related barriers	Knowledge gaps
		Socioeconomic barriers
10. Solutions to improve TUTT implementation	4.1 Expanding access through innovation	Alternative screening options
		Community-based approaches
	4.2 Education and capacity strengthening	Patient education and advocacy
		Provider training and role clarification

Summary of qualitative analysis categories, themes, and sub-themes derived from in-depth interviews with healthcare professionals on targeted universal TB testing (TUTT) implementation

### Category 1: The introduction of TUTT

#### Theme 1.1: Communication channels

Healthcare professionals described varied modes of TUTT introduction, ranging from ad hoc to structured processes. For some, information came informally, often as passing references in unrelated settings. One participant recalled:

*I first heard about TUTT in passing at a workshop on TB data analysis. There was no formal training*,* so I had to read up on it myself. Sometimes Operational Managers attend training*,* but information doesn’t always reach frontline staff (Nurse*,* Healthcare Professional [HP]_01).*

Another participant reflected:

*…from what I’ve seen*,* the introduction was largely through circulars from managers instructing healthcare workers on new policies. However*,* these instructions can be unclear*,* and healthcare workers often don’t receive detailed briefings or training. This approach makes implementation challenging* (Nurse, HP_02).

In contrast, structured communication and cascading training were seen as enabling smoother uptake. For example, a participant noted:

*TUTT was introduced as part of the National TB Recovery Plan in April 2022. We received a draft document from the DoH [Department of Health]*,* and after approval*,* we began working on implementation with guidance from provincial and district TB program leads. However*,* due to communication issues*,* it took time for full adoption. We eventually aligned our strategies by late 2023 through district and sub-district training sessions* (Program Manager, HP_03).

Others described district-led dissemination, mentoring, and integration into existing support visits and data review meetings, which helped clarify expectations and strengthen implementation. Several participants perceived that the clarity and mode of communication, whether through informal channels or structured dissemination, influenced how quickly and consistently TUTT was adopted. Facilities receiving clear guidance, training, and ongoing mentorship tended to implement the policy more effectively, while unclear or fragmented communication contributed to delays and inconsistent uptake.

#### Theme 1. 2: Capacity building

The availability and quality of training shaped early implementation experiences. Providers benefited from more structured support through district- and sub-district-level mentorship. One healthcare professional described how ongoing guidance complemented formal training gaps:

*The policy was shared through district channels*,* with periodic trainings and a focus on the TB Recovery Plan. My role includes mentoring facility clinicians to understand and implement TUTT*,* especially if they haven’t attended formal training. We also emphasize the policy in support visits and during facility data review meetings (Nurse*,* HP_05).*

In certain cases, locally driven initiatives served as a form of peer-led capacity building. For example:

*Targeted testing began in one facility where the OM [Operational Manager] initiated testing for high-risk groups. This successful approach expanded to the sub-district and eventually attracted provincial attention*,* leading to adoption across the area. When the National TB Recovery Plan was introduced*,* we were pleased to see our practices aligned with national policy (Program Manager*,* HP_04).*

Where mentorship and local leadership were present, practices were more likely to align with the TUTT policy intent, underscoring the value of sustained, hands-on guidance in building implementation capacity.

### Category 2: Feasibility of TUTT

#### Theme 2.1: Facility capacity

The feasibility of implementing TUTT was strongly shaped by facility capacity, particularly staffing levels, sputum collection logistics, and workflow organization. Participants working at facility level highlighted practical challenges such as managing all consultations with limited staff, difficulty in consistently collecting sputum, and patient reluctance to provide samples in high-volume settings. Two participants reflected:

*Testing everyone isn’t feasible in high-volume facilities due to the difficulty of sputum collection. Only a small percentage of clients come forward for TB testing after health education sessions*,* and logistics like sputum sample return rates make the process challenging* (Nurse, HP_01).

*In some facilities*,* a single clinician manages all patient consultations*,* making it difficult to consistently collect sputum samples. In others with more staff*,* the workflow runs more smoothly*,* allowing for complete TB and viral load testing in one visit* (Nurse, HP_06).

At the same time, facilities with stronger staffing capacity and clearer role distribution were able to align more closely with the TUTT policy intent:

*Yes*,* it’s feasible in facilities with adequate resources. However*,* some facilities face challenges like understaffing*,* which affects the quality of care. Facilities with strong staffing tend to show better data on annual TB testing for HIV-positive clients* (Nurse, HP_05).

Beyond staffing, feasibility was also linked to broader health system factors such as patient retention and laboratory support. Program managers, who operated at district and national level, emphasized that feasibility varied widely depending on available resources and management capacity:

*Annual testing is feasible*,* but issues like inadequate support for sputum collection and a shortage of dedicated staff create challenges. Operational Managers play a critical role in policy enforcement*,* but with multiple priorities*,* this often varies between facilities* (Program Manager, HP_03).

*Feasibility depends on retention in HIV care and facility resources. Patients are often lost to follow-up*,* impacting annual TB testing rates. Retention in HIV care and facility resources make a significant difference. Certain provinces*,* like KZN [KwaZulu-Natal]*,* manage better with budgeted resources*,* but other regions struggle due to funding limitations* (Program Manager, HP_08).

Taken together, the perspectives from both facility-level clinicians and program managers demonstrate that while TUTT was considered feasible in principle, its successful implementation was reliant on sufficient staffing, retention in HIV care, and district-level resource allocation.

### Theme 2.2: Data infrastructure

Participants emphasized that while existing platforms capture important program elements, the lack of integration between HIV and TB electronic data modules limits the ability to monitor TUTT effectively. While hosted on the same platform, the HIV and TB modules operate as standalone systems and are not linked at the individual level. The HIV module captures viral load testing well, while the TB module records the full diagnostic cascade; however, it is not possible to link viral load testing with the TB diagnostic cascade in real time at the health facility level. One participant explained:

*Yes*,* with the right data systems in place*,* TUTT can be highly effective. High coinfection rates among PWH in certain districts mean that targeted TB testing can yield great benefits. However*,* without robust data systems to track and account for testing*,* the program’s impact is harder to measure. Real-time data systems*,* which are currently lacking*,* could enhance monitoring and intervention* (Doctor, HP_10).

This data system fragmentation poses a particular challenge given that the TUTT policy recommends that annual TB testing for PWH be conducted alongside viral load monitoring. As another participant noted:

*Yes*,* it’s feasible. We have strong policy support*,* leadership*,* and laboratory capacity to exceed our annual testing targets. With the right data systems in place*,* TUTT could be effective. However*,* the integration of TB and HIV programs is challenging and requires coordinated efforts at the facility level* (Doctor, HP_07).

As a result, facilities are unable to track whether TB testing is systematically occurring at viral load visits, which constrains their ability to measure performance and identify missed opportunities. Strengthening data infrastructure, particularly through integrated, real-time systems, was therefore seen as central to improving TUTT’s operational feasibility, by enabling clinicians to follow patients more effectively, managers to identify drop-offs in real time, and TB and HIV programs to work in a more coordinated way.

### Category 3: TUTT implementation challenges

#### Theme 3.1: Operational constraints

Sputum collection was consistently described as a major operational barrier, particularly among asymptomatic PWH who struggled to produce a specimen during clinic visits, and in some cases, patients submitted saliva instead of sputum. Participants mentioned:

*Sputum collection is a huge hurdle*,* especially for asymptomatic patients*,* many of whom cannot produce a sample on-site. Additionally*,* giving patients bottles to take home often leads to poor sample quality or no sample return* (Nurse, HP_01).

*GXP [GeneXpert] testing at viral load appointments works well*,* but culture testing remains a challenge*,* often due to inadequate sputum collection from patients and inconsistent collection by staff* (Program Manager, HP_04).

The difficulty with sample collection was compounded for children, where healthcare professionals expressed a lack of confidence in performing gastric lavage. The challenge was not only technical but also structural, as limited staff capacity emerged from participant response:

*Challenges include limited staff capacity*,* inconsistent supervision*,* and the difficulty of obtaining sputum samples*,* especially from children* (Program Manager, HP_03).

### Theme 3.2: Patient-related barriers

Patient-related barriers included both knowledge gaps and socioeconomic challenges, which limited TUTT uptake and follow-through after testing. Several participants explained that asymptomatic patients were reluctant to test because they did not perceive themselves to be at risk of TB, while others who tested positive were sceptical of their diagnosis or did not accept treatment due to the absence of symptoms. Participants further highlighted that patients sometimes discontinue treatment when they feel better or experience side effects. Four participants reflected:

*Patients don’t always understand the need for testing unless they have symptoms. Therefore*,* we conduct health education sessions to inform them about TB symptoms and the need for testing*,* though turnout for testing isn’t always high* (Nurse, HP_01).

*Patients often don’t feel they need TB testing if they’re asymptomatic*,* and both adults and children struggle with sputum production. For children*,* there’s the added difficulty of non-invasive sample collection* (Nurse, HP_05).

*Other reasons include lack of knowledge; whereby asymptomatic patients often underestimate the seriousness of their condition and refuse to start TB treatment or silently transfer-out without notifying the facility* (Nurse, HP_06).

*Common reasons for patients not completing TB treatment include incorrect contact information and patients relocating. Some patients stop medication when they feel better*,* or if they experience side effects* (Program Manager, HP_03).

Socioeconomic barriers, including transport costs and competing livelihood priorities were also described. These barriers were not limited to TB testing but extended to broader healthcare access. For example, PWH often prioritized food or social support opportunities over attending clinic visits, resulting in missed opportunities to integrate annual TB testing within routine HIV care. One participant noted:

*Socioeconomic factors are a major issue. Many clients prioritize food over healthcare appointments*,* often skipping visits if community events offer food parcels or other basic necessities. This is a recurring theme*,* highlighting the need to integrate social support services* (Nurse, HP_09).

Taken together, these findings suggest that patient-level barriers to TUTT extend beyond initial testing to include reluctance to accept asymptomatic diagnoses, socioeconomic obstacles to retention, and systemic gaps that prevent timely initiation and completion of TB treatment.

### Category 4: Solutions to improve TUTT implementation

#### Theme 4.1: Expanding access through innovation

Participants highlighted the importance of diversifying diagnostic approaches to reduce reliance on sputum, which was consistently described as a barrier to effective TUTT implementation. Mobile digital chest X-rays were emphasized not as confirmatory tests but as valuable triage tools that could support both facility-based and community outreach services by identifying those at highest risk who should undergo confirmatory testing. Similarly, point-of-care C-reactive protein (CRP) testing was spontaneously suggested by participants as a rapid adjunct for prioritizing patients for further TB investigations, particularly in high-volume settings. These approaches were viewed as complementary to existing diagnostics, helping to streamline testing pathways and reduce missed opportunities. For example, two participants remarked:

*Point-of-care options like mobile chest X-rays could help identify TB cases without relying on sputum. Other tests*,* like CRP tests*,* could be beneficial*,* though they’re usually reserved for doctors* (Nurse, HP_01).

*CRP tests are helpful for quick assessments in high-volume settings*,* but we need consistent in-service education on new testing protocols* (Program Manager, HP_04).

Community-based approaches were repeatedly emphasized as integral to improving access, reducing stigma, and enhancing patient engagement. One participant explained:

*Adopting a patient-centred approach*,* where services are brought to key populations in their communities*,* would improve access. This would involve community-based screening and testing*,* rather than expecting them to visit facilities where they might face stigma. So*,* yes*,* I think bringing services into communities would improve access and reduce stigma* (Doctor, HP_10).

Another participant highlighted the potential of leveraging existing local structures and collaboration with other sectors:

*…using community platforms like “war rooms” [community-based service delivery platforms established to coordinate local government services and community engagement] to promote awareness*,* could enhance outcomes. Greater collaboration across sectors*,* such as with the Department of Education*,* could support improved TB outcomes for all age groups *(Program Manager, HP_03).

These strategies underscore the value of meeting patients where they are, both geographically and socially, to strengthen uptake of TB testing.

### Theme 4.2: Education and capacity strengthening

Participants emphasized that both patient and provider education were central to strengthening TUTT implementation. Limited awareness among patients, particularly those who were asymptomatic, often reduced uptake of TB testing. As one participant explained:

*Health education is key. When patients understand the reasons behind testing*,* they’re more cooperative *(Nurse, HP_05).

Similarly, another participant stressed that adopting educational strategies modelled on existing HIV programs could enhance acceptance of TB testing and preventive therapy:

*Adopting educational strategies like those in the HIV program would make a difference. Educating patients on TB*,* TPT*,* and medication adherence could improve understanding and acceptance. We could also address pill burden by emphasizing the importance of TPT for TB prevention. Leveraging the well-established HIV support structure could provide the framework needed to improve TB support* (Program Manager, HP_11).

On the provider side, training, mentorship, and clear role delineation were repeatedly identified as essential for consistent and high-quality implementation. Many participants highlighted the heavy workload and role overlap between cadres such as lay counsellors and nurses, which created inefficiencies and gaps in testing. One participant suggested:

*Defining staff roles and empowering patients to ask for TB testing could help. Many lay counsellors feel overburdened*,* doing tasks that nurses are too busy to handle. Clearer role assignments and a better distribution of work could improve implementation* (Nurse, HP_02).

Similarly, targeted training in technical skills was viewed as critical, particularly for paediatric TB diagnosis. As one participant noted:

*For children testing*,* we plan to collaborate with physiotherapists to train staff in collecting gastric washouts. Involving OMs [Operational Managers] more actively could also improve supervision for quality sputum samples* (Program Manager, HP_04).

Together, these perspectives underscore that strengthening TUTT requires not only improving patient awareness and demand, but also equipping healthcare providers with the skills, mentorship, and clarity needed to deliver testing consistently and effectively.

## Discussion

We explored healthcare professionals’ perceptions of the introduction, feasibility, challenges, and potential solutions for implementing TUTT. The introduction of TUTT was characterised by varied communication approaches, ranging from informal and inconsistent messaging to coordinated dissemination through formal channels, with program managers and facility-based staff often reporting different experiences. Access to training and mentorship was uneven, with gaps in structured support contrasting with facilities where mentoring and role clarification aided adoption. The feasibility of TUTT was influenced by staffing capacity, diagnostic resources, workflow organisation, and the integration of TB and HIV data systems, which remain fragmented. Perceived implementation challenges included operational constraints such as heavy workloads, staff shortages, and persistent difficulties in sputum collection, particularly among asymptomatic PWH. Patient engagement was further hindered by limited awareness of TUTT and socioeconomic barriers. Proposed solutions emphasised expanding access through triage tools such as mobile digital chest X-rays and point-of-care CRP testing to better identify those at highest risk, alongside community-based outreach and strengthened patient education, provider training, and role clarity.

Our findings indicate that TUTT was widely perceived as a valuable strategy for identifying individuals with undiagnosed TB. However, its introduction through a predominantly top-down approach resulted in inconsistent communication and variable adoption [9]. Top-down approaches, characterised by centralized decision-making, prioritize national-level objectives while leaving operational details unresolved [17, 18]. In contrast, bottom-up approaches leverage local autonomy and contextual insights of frontline implementers to adapt policies more effectively [17, 18]. In the absence of clear, standardized communication and feedback mechanisms, frontline providers were required to interpret and operationalise TUTT independently, contributing to deviations from policy intent and reduced implementation fidelity [9]. Some participants in our study even reported different timelines for when TUTT began, reflecting variations in how provinces and districts operationalized the policy and when frontline staff were engaged. An integrated approach combining national-level guidelines and resources with local adaptation, piloting, and structured feedback, could enhance consistency and responsiveness, facilitating more equitable and effective policy implementation [10, 18].

Structured workshops and training sessions provided a more cohesive framework for implementing TUTT; however, access to these opportunities varied widely across districts and facilities. Such disparities are linked to logistical barriers such as limited training budgets, scheduling conflicts, and training workforce shortages [19]. Similar challenges have been reported elsewhere, where inadequate or inconsistent training undermined provider capacity to deliver high-quality TB services [20]. A systematic review also confirmed that inequitable training access in low- and middle-income countries is frequently linked to resource constraints and workforce shortages [21]. These gaps may compound challenges of top-down rollouts, leaving frontline implementers to interpret and operationalize new policies without adequate preparation [17].

Heavy workloads, staff shortages, and resource constraints were central to healthcare professionals’ perceptions of the feasibility of TUTT. Facilities with adequate staffing, organized workflows, and reliable supply chains were perceived as better positioned to integrate TB testing into HIV care, while under-resourced facilities struggled to maintain consistency. Similar findings have been reported in other TB and HIV programme evaluations, where increased workloads and inadequate resources undermine policy implementation fidelity [21, 22]. For TUTT, addressing these constraints will require equitable resource allocation, continuous performance monitoring, and site-specific problem-solving.

Reliable data systems were viewed as essential for both implementing and evaluating TUTT. Participants noted that although the current national electronic register captures TB and HIV program data, limited interoperability between the two modules prevents facilities from automatically linking TB testing with viral load visits, limiting performance monitoring. Similar challenges have been reported in modifying entrenched systems, often constrained by outdated platforms and slow procurement processes [23, 24]. Strengthening TUTT will require investment in interoperable, integrated surveillance systems and additional fields to capture TB testing at viral load visits, consistent with WHO recommendations [23, 25, 26].

Sputum-based testing posed operational difficulties, particularly for asymptomatic PWH. While routine care settings report frequent challenges, research contexts often achieve higher sputum collection rates [27]. This contrast likely reflects not only differences in provider motivation, patient engagement, and support mechanisms, but also structural factors such as smaller patient volumes per provider and greater time available to support sputum production in research settings [27, 28]. These findings highlight the need for targeted investment in healthcare worker training, protected time, and dedicated resources to support sputum quality and collection techniques within busy clinics, rather than assuming sputum collection failure is inevitable. Understanding these contextual factors could inform strategies to improve sputum collection in routine care, reduce reliance on a single diagnostic pathway, and explore the promise of non-sputum diagnostic methods such as oral/tongue swabs as complementary options [4, 28–30].

Challenges in patient engagement, including knowledge gaps and socioeconomic barriers, are closely linked to broader determinants of health such as limited access to healthcare services, transportation difficulties, and low health literacy [31, 32]. These factors often lead patients, particularly those without symptoms, to deprioritize TB testing, a phenomenon well-documented in public health literature [33]. Addressing these barriers requires more than health system–driven interventions; it calls for robust multisectoral collaboration to tackle the structural and socioeconomic inequities that disproportionately affect vulnerable populations [32].

Evidence suggests that facility-based strategies such as TUTT can capitalize on routine HIV visits to efficiently identify high-risk individuals already engaged in care [11]. Where structural barriers limit access to health facilities, community-based strategies like mobile screening units can extend reach and mitigate stigma, though these approaches are resource-intensive and best used as complements rather than the core of TUTT [34, 35]. Complementary health education and patient-centred approaches strengthen awareness, improve acceptance, and increase participation in TB screening [20, 36]. Integrating patient education with facility-based TUTT, while selectively deploying community outreach, may facilitate earlier diagnosis, strengthen linkage to care, and improve TUTT implementation outcomes [37].

Our study has several limitations. First, the qualitative design and purposive sampling provide contextual insights rather than broad generalizability across all districts and facilities. Nonetheless, the diverse perspectives of nurses, doctors, and program managers strengthen the relevance of the themes identified. Second, participant responses may have been subject to recall bias or social desirability bias, potentially influencing the accuracy and authenticity of the information provided. Third, the focus on healthcare professionals’ perspectives excludes the views of other critical stakeholders, such as patients, whose experiences could provide a more comprehensive understanding of TUTT implementation. Finally, while participants had substantial experience with TUTT at the time of interviews, the study did not capture long-term outcomes, limiting conclusions about sustainability, scalability, and ultimate impact on TB detection and treatment.

Despite these limitations, our findings have important implications for strengthening TUTT and TB case finding among PWH. Addressing staff shortages and workflow gaps is essential to improve feasibility and fidelity of implementation [7, 9]. Structured training and mentorship can enhance the integration of TB and HIV services at facility level, while upgraded information systems linking TB and HIV modules are critical for real-time monitoring and evaluation of TUTT outcomes [12, 24]. Community-based approaches, such as mobile screening units, decentralized sample collection, and patient-centred health education, can help overcome socioeconomic and access-related barriers, supporting earlier diagnosis, linkage to care, and treatment initiation [34, 36]. Collectively, these interventions highlight the need for a comprehensive approach that combines resource investment, capacity building, and innovative service delivery models to strengthen TB control [34].

To build on these findings, future research should address key gaps identified in this study, particularly, persistent resource constraints, fragmented TB/HIV data systems, uneven access to provider training, and patient-related barriers such as low awareness of TUTT and socioeconomic barriers. Expanding the scope to include patients, community members, and other relevant stakeholders would provide a more holistic understanding of TUTT implementation challenges and opportunities. Evaluating integrated TB/HIV data systems is needed to strengthen routine program monitoring. Longitudinal studies are also needed to assess the long-term impact of TUTT on TB detection, treatment outcomes, and health system performance. Generating this evidence will be essential for refining policies and ensuring that TUTT fulfils its potential as a targeted yet universal strategy to reduce TB morbidity and mortality among PWH.

## Conclusion

This study underscores that while TUTT is widely perceived as a valuable strategy for enhancing TB detection among PWH, its success depends on more than policy endorsement. Gaps between national policy intent and facility-level implementation, driven largely by top-down rollout and variable communication, undermined implementation fidelity across settings. Effective implementation requires clear and consistent communication during policy rollout, alongside a combination of adequate resources, responsive data systems, strong provider capacity, including clear delineation of roles and responsibilities among frontline staff such as nurses, lay counsellors, and mentors, and patient-centred, community-driven approaches. Addressing gaps across the full implementation pathway, from introduction and feasibility to operational challenges and practical solutions, through targeted investment in workforce capacity, interoperable data systems, and sustained implementation support will enable TUTT to move from a promising policy to a consistently applied practice capable of accelerating progress towards TB elimination goals.

## Supplementary Information

Below is the link to the electronic supplementary material.

Supplementary Material 1

## Data Availability

The datasets generated and/or analysed are not publicly available but can be made available on reasonable request to the authors and subject to approval by the University of Cape Town Human Research Ethics Committee.

## Abbreviations

AIDS: Acquired Immunodeficiency Syndrome
CRP: C-reactive Protein
HIV: Human Immunodeficiency Virus
HP: Healthcare Professional
PEPFAR: U.S. President’s Emergency Plan for AIDS Relief
PWH: People with HIV
TB: Tuberculosis
TUTT: Targeted Universal Tuberculosis Testing
WHO: World Health Organization
