# Supplementary material for: Feasibility, challenges, and solutions for implementing targeted universal tuberculosis testing: perceptions of healthcare professionals in South Africa
Source: BMC Health Serv Res. 2026 Feb 14;26:383. doi: 10.1186/s12913-026-14156-3 (PMC13011570; doi:10.1186/s12913-026-14156-3)
Supplement: Supplementary file 1 — Supplementary Material 1 [file 12913_2026_14156_MOESM1_ESM.pdf]

Healthcare Professionals In-Depth Interview Guide

Title: Feasibility, challenges, and solutions for implementing targeted universal tuberculosis testing: Perceptions of healthcare professionals in South Africa

Interview date: ___________

Type of interview (mark with X) In-person ___ Virtual ___

Interview start time ___: ___ Interview end time ___: ___

Study ID: _______________

1. Can you tell us more about your role in providing TB/HIV integrated services?
    - How long have you worked in this role?

2. Can you tell us how big is the problem with TB among people with HIV (PWH) in your province/district/facility?

3. Can you tell us about the current approach used in your province/district/facility to screen and test PWH for TB?

4. Please share with us how the targeted universal TB testing (TUTT) policy was introduced in your province/district/facility?

5. Can you tell us about the process of ensuring that PWH that are already on ART are tested for TB annually according to TUTT?
    - Do you think this process is feasible?
        - Why / why not?

6. What are some of the challenges you experience with screening and testing PWH for TB as outlined in the TUTT policy?
    - What do you think would make it easier to overcome these challenges?

7. Can you tell us more about the processes in your province/district/facility used to review and monitor performance on TB screening, testing, and treatment initiation?

8. What are some of the challenges PWH have with being screened and tested for TB?
    - For those that are diagnosed with TB, what are their challenges with starting and completing TB treatment?

9. What do you think could be done to address these challenges?

10. Are there any challenges you particularly faced that you would like to share with us?
    - Hints: coordination, staffing, training, lack of resources, guidelines

11. Based on your personal experience in the TB/HIV field, are there any recommendations you would like to make to improve TB case finding in PWH?
    - Hints: recommendation for PWH with presumptive or confirmed TB, district support partners, national TB and HIV programs and TB donor agencies, etc.

We have reached the end of our interview. Thank you for your participation.
